# Predicting Cross-Reactivity and Antigen Specificity of T Cell Receptors

**DOI:** 10.3389/fimmu.2020.565096

**Published:** 2020-10-22

**Authors:** Chloe H. Lee, Mariolina Salio, Giorgio Napolitani, Graham Ogg, Alison Simmons, Hashem Koohy

**Affiliations:** ^1^MRC Human Immunology Unit, Medical Research Council (MRC) Weatherall Institute of Molecular Medicine (WIMM), John Radcliffe Hospital, University of Oxford, Oxford, United Kingdom; ^2^MRC WIMM Centre for Computational Biology, Medical Research Council (MRC) Weatherall Institute of Molecular Medicine, John Radcliffe Hospital, University of Oxford, Oxford, United Kingdom; ^3^Translational Gastroenterology Unit, John Radcliffe Hospital, Oxford, United Kingdom

**Keywords:** T cell cross reactivity, T cell specificity, epitope, antigen presentation, adaptive immune system, antigen specificity

## Abstract

Adaptive immune recognition is mediated by specific interactions between heterodimeric T cell receptors (TCRs) and their cognate peptide-MHC (pMHC) ligands, and the methods to accurately predict TCR:pMHC interaction would have profound clinical, therapeutic and pharmaceutical applications. Herein, we review recent developments in predicting cross-reactivity and antigen specificity of TCR recognition. We discuss current experimental and computational approaches to investigate cross-reactivity and antigen-specificity of TCRs and highlight how integrating kinetic, biophysical and structural features may offer valuable insights in modeling immunogenicity. We further underscore the close inter-relationship of these two interconnected notions and the need to investigate each in the light of the other for a better understanding of T cell responsiveness for the effective clinical applications.

## Introduction

Specific molecular interactions between heterodimeric T cell receptors (TCRs) and their cognate peptide-MHC (pMHC) ligands contribute to the nature of ensuing adaptive immune response. A better understanding of TCR:pMHC interaction is required to be able to harness adaptive T cell immunity effectively for vaccines and therapeutics. Unfortunately, the mechanisms underpinning cross-reactivity and antigen specificity of peptide-specific TCRs remain puzzling, and leaves the community with an incomplete picture of T cell recognition.

Cross-reactivity is defined as the capacity of a TCR to recognize more than one peptide-MHC molecule. The idea was first postulated by Matzinger and Bevan (1) and later gained traction via Don Mason who challenged the dominant clonal selection theory arguing a highly incompetent immune system if a TCR was able to recognize only a single pMHC complex (2), and Andrew Sewell who empirically measured the necessity of cross-reactivity given the insufficient number of TCRαβ to protect against a wide spectrum of pathogen by comparing the number of potential foreign pMHC complexes a T cell might encounter and the number of TCRs available (3).

Although it is known that T cells can recognize peptide and non-peptide antigens, it is now well-accepted that peptide-specific TCRs exhibit high levels of cross-reactivity. In fact, it has been proposed that a single TCR can recognize 10^4^-10^7^ different MHC-associated epitopes (2). However, it has also been illustrated that once a TCR reacts with a specific peptide-MHC complex, the probability of it reacting with another randomly chosen peptide reduces to ~10^−4^ (4). Thus, TCR recognition of pMHC complexes is both cross-reactive, given the high number of total epitopes that could be bound, and at the same time, highly specific considering the low frequency of epitopes that can be recognized by a given TCR.

Recent biological and computational advances to screen antigenic peptides and profile TCR repertoires have greatly improved our understanding of the TCR:pMHC interaction. However, the picture is far from complete. As yet it is not possible to, (a) predict TCRs recognizing a given antigen, or (b) predict antigens recognized by a given TCR. Methods to accurately predict biological specificity or cross-reactivity would have profound clinical, therapeutic and pharmaceutical applications in designing cellular therapies for fighting cancer, autoimmune and infectious diseases.

New biological methodologies have enabled definition of cross-reactive peptides using high throughput screens against a series of TCR molecules and some can screen whole cells (5–14). In fact, recently developed labeled pMHC multimers coupled with single cell sequencing facilitated screening of an array of immunogenic peptides in a relatively high-throughput manner, but this is still limited by practical synthesis of pMHC multimers (6). Additionally, kinetic, biophysical and structural studies provide insights on the complex landscape of TCR:pMHC interaction from different angles. However, apart from few attempts to predict immunogenicity, algorithms to predict breadth and/or constituents of the cross-reactome are still in their infancy.

Understanding the underlying mechanisms of common antigen specificity of TCRs, on the other hand, has been the focus of key research over the past few years. A number of recent studies have demonstrated the plausibility of identifying shared motifs amongst tetramer-specific TCRs (15–18) to explain shared antigen specificity. By the advancement of high throughput sequencing technologies for both bulk and single cell immune repertoire profiling, lack of biological data is becoming less of a challenge, allowing us to simultaneously project TCR profiles of T cells along with their antigen specificity, functional states and gene expression levels (19, 20). Therefore, part of the challenge turns into developing sophisticated mathematical and computational models to understand high dimensional and occasionally sparse datasets.

Although cross-reactivity and common antigen-specificity have been investigated individually, understanding the relationship of the two closely interconnected notions seems to be underrepresented in the research community. Whereas, in order to set a foundation for better understanding T cell responsiveness for effective clinical applications, these two pillars of the adaptive immunity can be and should be investigated together and each in the light of the other.

Here we review the recent advances in the understanding of both cross-reactivity and common specificity of T cell recognition mainly from a computational perspective. We will discuss current experimental and computational approaches to investigate cross-reactivity, and highlight how integrating kinetic, biophysical and structural features may offer valuable insights in modeling immunogenicity against TCRs. We will then discuss the progress and limitations in assigning antigen-specific TCRs based on their shared features. Lastly, we will underscore the close inter-relationship of these two principles and how recent single cell technologies are poised to shed further in this area.

## Predicting Cross-Recognition Potential of T Cell Receptors

### Cross-Reactivity of TCR Is a Double-Edged Sword

TCR cross-reactivity, which was coined in the late twentieth and early twenty first century, has become recognized as a common feature of TCR recognition (2, 3, 21–23) and a single TCR is estimated to bind >10^6^ different MHC-bound peptides (24). A repertoire of highly cross-reactive T cells can effectively screen for numerous antigenic peptides and minimize the risk of pathogen escape from immune surveillance. Nonetheless, cross-reactivity is a double-edged sword: while a highly cross-reactive T cell can effectively screen for a wide spectrum of epitopes, this may also lead to dysregulated T cell responses potentially contributing to allergy, immunopathology, autoimmunity and chronic infection (25–28).

Prior exposure of degenerate T cells can induce polarized response to a pathogen or vaccination (29, 30). Heterologous immunity has been reported between related pathogens with high sequence similarity as well as unrelated pathogens with minimal sequence overlap (31–35) giving both positive and negative effects (36, 37). There is accumulating evidence that genetic background, private TCR specificities and immunological history are key factors contributing to the final outcome of antigen exposure—whether to confer protective immunity or induce damaging immunopathology (25, 38, 39). It is of note that peptide recognition is not a simple on/off event, and that the same T cell can respond in different ways to modified peptides, by for example, pMHC affinity and dose thresholds (40, 41), co-stimulatory molecules (42), and hierarchical organization of thresholds (43, 44).

While naïve T cells expressing self-reactive TCRs survive due to the low avidity or low expression of peptides derived from self-proteins (45), immune tolerance may be broken if T cells are activated by cross-recognition of pathogenic peptides. This results in memory T cells that are potentially stimulated even at 50x lower peptide concentrations (46, 47). Such a phenomenon, known as molecular mimicry, may occur via induced fitting by TCR or pMHC, altered TCR:pMHC docking geometry, and/or structural degeneracy leading to cross-recognition of low-affinity TCR:pMHC, thus potentially leading to breakdown of tolerance (48–53).

From a clinical perspective, recent immunotherapy trials have highlighted off-target toxicities triggered by cross-reactivity of high affinity TCRs, where adoptive T cell transfer trials with high-avidity DMF5 TCR targeting the HLA-A^*^02:01 MART-1 melanoma peptide showed a greater promise than DMF4 TCR for cancer treatment but also triggered autotoxicities (54). In addition, adoptive T cell transfer targeting melanoma-associated antigen 3 (MAGE-A3) peptide demonstrated severe cardiac toxicity, attributed to recognition of unrelated peptide derived from self-protein Titin displayed on HLA-A^*^01:01 on the surface of healthy cardiac cells (55). This seemingly unpredictable off-target toxicity mediated by T cell cross-reactivity highlights the requirement to mitigate against autoimmunity deriving from TCR-based immunotherapy.

### Technologies to Elucidate the Landscape of Cross-Reactivity

Inability to detect potential toxicities through initial safety evaluation highlighted the need to develop technologies to assess the cross-recognition potential of each TCRs engineered for clinical uses. In recent years, technologies to extensively characterize the recognition pattern of TCR:pMHC have emerged [reviewed in (56)].

Briefly, large combinatorial peptide libraries (8, 9, 24) along with peptide-MHC display systems (7, 10–13) enabled unbiased screening of pooled pMHCs against TCRs to determine positional amino acid preferences. Combinatorial peptide libraries containing thousands to millions of peptides have been utilized to identify cognate peptides, estimate the cross-recognition potential of TCRs, and further characterize structural and/or biochemical relatedness between peptides recognized by the same TCR (10, 57–60).

With the help of combinatorial peptide libraries and single amino acid analogs, the “hotspots” crucial for potential off-target cross-reactivity have been characterized (61–63). For instance, Border et al. demonstrated the capability of single amino acid analogs (called “X-scans”) to differentiate cross-recognition potential of two affinity-enhanced TCRs which would otherwise appear similarly potent and specific (64).

While binding of recombinant TCR and pMHC molecules provide essential information, previous studies reported high-affinity, yet non-stimulatory, interactions occur with high frequency in the human T cell repertoire (65, 66). In recent years, several cell-based platforms have been developed for TCR antigen discovery, using T cell clones or TCR-transduced T cells, for a better reflection of *in vivo* systems without requirement for a soluble TCR (7, 11–14). One example is signaling and antigen-presenting bifunctional receptors (SABRs), where a signaling domain has been introduced to in the MHC-I molecule, leading to green fluorescence protein (GFP) expression following TCR:pMHC interaction (11). Compared to conventional yeast display system, these approaches enabled a rapid identification of antigens expressed from large peptide libraries transduced into the target cells. However, there remains a number of limitations.

### Limitations of the Current Technologies

Although recent approaches provide increased flexibility to investigate the degeneracy of TCRs, they remain limited in (i) the number of possible TCRs that can be tested against peptide libraries in a single experiment, (ii) the number of peptides compared to the actual number of ligands that might be encountered, (iii) the need to prepare a new peptide library for each analysis of pMHC specificity, (iv) the high number of false positive and negative peptides resulted from screening, and (v) often the requirement to generate individual recombinant TCR, T cell clones, or reporter cells expressing TCR for screening. Some approaches in ongoing development do offer the potential to obtain high-throughput biological data using primary unmodified polyclonal T cells (7).

Moreover, current strategies of generating a single amino acid analog library rely on replacing a pre-established peptide target with one amino acid at a time. However, such an approach may underscore the possibility of duplex or triplex amino acid substitutions or even largely different peptides to trigger a TCR response (67). Therefore, interpretation of the results should reflect that it may merely be a window of estimated cross-reactivity.

### Expanding Knowledge of TCR:pMHC Interactions by *in silico* Modeling

*In silico* modeling may enhance the utility of experimental data for assessing TCR binding degeneracy. Associating the information gained from the aforementioned technologies with the knowledge of the human proteome and the HLA presentation potential through implementation of mathematical modeling approaches might provide valuable insights on the relationship between antigen specificity and cross-recognition potential of TCRs.

Moreover, *in silico* investigations may suggest clues to yet unsolved problems and help define how ubiquitous previously observed phenomena are, such as publicness of cross-reactive TCRs, different extent of cross-reactivity between featured and featureless peptides, the role of dominant peptides in TCR repertoire organization and preferential directionality of antigen specificity.

For example, Kasprowicz et al. observed preferential directionality from Hepatitis C Virus (HCV) to Influenza A Virus (IAV) i.e., a T cell primed with an HCV-derived peptide was capable of recognizing an IAV-derived peptide but the opposite was not true (68). Correspondingly, recent studies suggest that heterologous immunity is greatly influenced by private specificities and immunological history (39, 69, 70). However, due to scarcity of data and cost associated with generating the data, it is difficult to assay the prevalence and understand the underlying principle of antigen-driven repertoire convergence in an experimental setup. In this regard, *in silico* approaches may be more suitable for identifying patterns and testing hypotheses on factors driving observed phenomena.

Indeed, several groups have started to use modeling approaches to test various hypotheses on TCR:pMHC interaction propensities (38, 71, 72). For instance, Xu and Jo utilized a simple string model to evaluate a trade-off between rapid screening and dissociation penalty, and have shown that while a highly cross-reactive TCR detects correct peptides in a short period of time with the help of its degeneracy, it takes much longer to release from an incorrectly bound peptide (71).

In addition to models predicting TCR:pMHC interactions, models to relate TCR:pMHC binding parameters and antigen doses to T cell response have also been proposed [reviewed in (73)]. Recently, Fernandes et al. utilized partial differential equations to study the underlying mechanism of ligand discrimination and TCR triggering based on two physical properties, (i) TCR dwell time in the absence of large tyrosine phosphatase, and (ii) spatial constraints on the contact area, and found that topographically constrained T cell contacts allow, and may even be essential, for ligand discrimination by T cells (72). Although these mathematical models are built upon underlying assumptions e.g., a positive correlation between binding affinity and the extent of TCR cross-reactivity, provided that assumptions are evidence-based and reasonable, such modeling approaches will be a valuable strategy to quickly test hypothesis on cross-recognition potential.

### Approaches to Predict Immunogenicity From Experimental Data

In 1998, Don Mason argued in favor of the necessity for cross-reactivity (2) with an incredibly high number of peptides potentially generated from the 20 amino acids (>10^18^ peptides) and a relatively limited number of unique TCRs in an individual (in the range of 10^6^-10^8^) (3, 74, 75). Moreover, the possibility of post-translational modification, peptide processing, HLA presentation and altered T cell functions attributes additional factors to deciphering T cell targets (76–82).

Several attempts to estimate the polyspecificity of TCRs have been performed. These include: (i) generation of mutant peptides with amino acid substitutions and testing the impact of substitution on T cell activation and/or cytotoxicity (83–85), (ii) scanning combinatorial peptide libraries to find cross-reactive peptides against a TCR of known antigen specificity (24, 57, 59, 60, 86, 87), and (iii) scanning peptides or pMHC multimers derived from the host (e.g., human) or pathogen proteome to test cross-recognition potential (5–7, 88–92). Although functional readouts may not have captured all binding, the readout from these approaches allowed identification of essential interaction residues in TCR:pMHC, which were applied to predict polyspecificity.

For instance, in a recent TCR fingerprinting study, Karapetyan et al. investigated which amino acids at each position are essential for 1G4 TCR binding, activation and killing by sequentially replacing every amino acid position outside of anchor positions 2 and 9 with 19 alternative amino acids. The peptides were analyzed using three *in vitro* assays examining binding of NY-ESO^c259^ TCR to peptide-MHC complexes, activation of TCR-expressing cells and killing of target cells. Based on the experimental measurements, they constructed positional weight matrices (PWMs) for three *in vitro* assays and utilized PWM-defined kernel along with NetMHCpan v3.0, an algorithm to predict MHC binding, to predict peptides with high TCR recognition score. By applying the algorithm to 336,921 predicted HLA-A*02:01 binding 9-mer peptides, they demonstrated a strong activation of primary T cells out of the top scoring peptides.

Instead of scanning a single TCR, a few algorithms have been designed to predict immunogenicity of a peptide against a pool of TCRs by the use of sequences (93), positional information (94, 95) and/or physicochemical properties (96, 97) (Table 1). Pogorelyy et al. classified immunogenic vs. non-immunogenic peptides in Kidera feature space by transforming epitope sequences into a vector of sum for each of 10 Kidera factors that encode physicochemical properties of amino acids. Similarly, Ogishi et al. incorporated Amino Acid index database (AAindex) (110) and other physicochemical properties describing features determinant of immunogenicity, then compressed the most predictive peptide descriptors and contact potential profiling (CPP)-based features into a linear coordinate system through a machine-learning technique known as Extremely Randomized Tree (ERT) algorithm (96). Of interest, they hypothesized that immunodominant epitopes share intrinsic patterns which render them more prone to be recognized by the immune system of multiple individuals and focused on identifying these prominent features.

**Table 1:** List of algorithms to predict immunogenicity.

**References**	**Training data**	**Algorithm**	**Discriminative features (Immunogenicity)**
Tung et al. (95)	Trained on 9-mer HLA-A2 restricted peptides. From MHCPEP, SYFPEITHI and IEDB, consist of 558 immunogenic, 527 non-immunogenic peptides	Decision tree learning methods to identify informative physicochemical properties from 531 physicochemical properties retrieved from version 9.0 of amino acid index (AAindex) database. Support vector machine with a weighted string kernels for immunogenicity prediction (named POPISK)	Top AAindex contributors: (i) Retention coefficient in HPLC, pH2.1, (ii) Principal property value z2, (iii) Hydrophobicity scale from native proteins, (iv) Normalized composition of membrane proteins, and (v) pK-C. Found positions 4, 6, 8, and 9 critical for 9-mer peptide
Calis et al. (98)	Trained on 9-mer from MHC-I associated peptides. From IEDB and three immunogenicity studies in mice (99, 100), and unpublished data on Coxiella Burnetti-derived peptides), consist of 600 immunogenic and 181 non-immunogenic peptides	Per non-anchor residue of the presented peptide, log enrichment score calculated as ratio between the fraction of specific amino acid in immunogenic vs. non-immunogenic data, then score weighted to the importance of that position measured as Kullback-Leibler divergence. The weighted log enrichment scores of all (non-anchor) residues summed as immunogenicity score	Preference for residues with larger or aromatic side chains Positions 4–6 critical for 9-mer peptide
Trolle and Nielsen (101)	Trained on 9-mer peptides covering 9 HLA alleles. From 295 T cell epitopes from SYFPEITHI and 1,216 T cell epitopes from IEDB, allele-balanced training data created by randomly selecting 50 epitopes from each of 9 HLA alleles except 2 alleles having 14 epitopes each, Total 378 epitopes	Weighted sum of pMHC binding affinity [NetMHCcons (102)], pMHC stability [NetMHCstab (103)] and T cell propensity prediction (98) (integrated algorithm named NetTepi). Optimal relative weights obtained	Performance gain obtained by summing pMHC binding affinity, pMHC stability predictions and T cell propensity than individual predictions
Chowell et al. (104)	Trained on 9-mer H-2D^b^ and HLA-A2 restricted peptides (separately for two ANN-Hydro models). From IEDB, 204 immunogenic and 232 non-immunogenic (self-peptides from MHC ligand elution experiment with no known immunogenicity) for H-2D^b^, and 372 immunogenic and 201 non-immunogenic peptides for HLA-A2	Hydrophobicity-based artificial neural network (ANN-Hydro) based on numeric sequence of amino acid hydrophobicity	Strong bias toward hydrophobic amino acids at TCR contact residues (P4, P6, P7, and P8 for 9-mers) within immunogenic epitopes. Negative correlation between polarity of amino acids and immunogenicity
Łuksza et al. (105)	Trained on 2,552 MHC-I immunogenic peptides from IEDB. Neoantigens with mutations generated from non-hydrophobic, wild-type residues at positions 2 and 9 excluded (as prediction of MHC affinities for wild-type peptides with non-hydrophobic anchor residues led to non-informative amplitudes)	Recognition potential of a neoantigen = A × R, where amplitude (A) is relative probability that a neoantigen is presented on MHC-I whereas its wild-type counterpart is not, and R is probability that neoantigen will be recognized by TCR repertoire. R defined by a multistate thermodynamic model, treating sequence similarity as proxy for binding energies	High sequence similarity of a given neoantigen with epitopes in IEDB by gapless alignment with BLOSUM62 amino acid similarity matrix
Bjerregaard et al. (106)	From 13 publications, analyzed total 1,948 peptide-HLA complexes, of which 53 reported immunogenic	HLA binding prediction by NetMHCpan-4.0. Similarity between each neo- and normal peptide using kernel similarity measure proposed by Shen et al. (107)	High predicted binding score (HLA binding strength). Peptide sequence dissimilarity to self (wild-type counterpart of the neopeptide), especially for those with comparable HLA binding
Pogorelyy et al. (97)	Trained on 9-mer peptides. From (104), 3,671 immunogenic and 3,911 non-immunogenic peptides	Principal component analysis and dimensionality reduction on 10-dimensional vectors of Kidera factor sums for each epitope. Fit multinomial Gaussian model using expectation maximization to estimate probability of being immunogenic	Distinct physicochemical properties in Kidera space
Jurtz et al. (93)	Trained on 8,920 TCRβ CDR3 sequences and 91 HLA-A2 cognate peptides obtained from IEDB. 379 TCR and 16 peptides from the MIRA assay in (108). Negative data from eluted peptide ligands from self (i.e., human) proteins, a set of 200,000 TCR CDR3 sequences from 20 healthy donors and creating internal incorrect combinations of TCRs and peptides	Convolutional neural networks (CNN) to predict whether a given TCR is able to recognize a specific peptide, with amino acid sequences of peptide and CDR3 region of TCRβ chain as input. CNNs scans the input and detects pattern to be integrated into network (named NetTCR)	Conserved sequence patterns of peptide-TCR pairs encoded by BLOSUM50 matrix
Smith et al. (94)	Trained on 8-11mer 141 epitopes from MHC-I H2^b^ and H2^d^ haplotypes	Using amino acid features (tiny, small, aliphatic, aromatic, non-polar, polar, charged, basic and acidic), variables derived by presence/absence of each feature at each absolute and relative position, at site of SNV mutation, at being/middle/end residues, difference of each feature in mutated vs. reference antigen. Most predictive features into gradient boosting algorithm and trained by 10,000-fold cross-validation	Peptide biochemical features: valine at position 1, valine at last position, small amino acids at the last position, basic amino acids of the reference at the mutated position, changes in the mutated position to a small amino acid, lysine at relative site 1, and presence of valine within the first 3 positions
Ogishi and Yotsuyanagi (96)	Trained on 8–11 mer MHC-I and 11–30 mer MHC-II peptides. From IEDB, LANL HIV and HCV database and TANTIGEN database, 6,957 HLA-I and 16,642 HLA-II immunogenic peptides. 191,326 TCR CDR3β sequences obtained from MiXCR	TCR-peptide contact potential profiling (CPP) by optimal alignment between CDR3β (randomly down-sampled to 10,000 sequences) and peptides and using pairwise contact potential scales from AAindex. Peptide sequence-based estimates of physicochemical properties (= peptide descriptors) using: *aIndex, blosumIndices, boman, charge, crucianProperties, fasgaiVectors, hmoment, hydrophobicity, instaIndex, kideraFactors, mswhimScores, pI, protFP, chseScales, and zScales* Most predictive peptide descriptors and CPP features compressed into a linear coordinate system through extremely randomized tree (ERT) algorithm	Physicochemical and CPP features: features from short (3- and 4-aa) and longest (8- and 11-aa for MHC-I and MHC-II, respectively) fragments, skewness- and kurtosis-derived features and AAindexes, including inverse of modified Miyazawa-Jernigan transfer energy, inverse of quasichemical energy in an average protein environment from interfacial regions of protein-protein complexes, and distance-dependent statistical potential within 10–12 Å
Riley et al. (109)	Trained on 9-mer HLA-A2 restricted peptides. 155 immunogenic from IEDB, 2,756 HeLa HLA-A2 binding self-peptides and 1,044 HLA-A2 non-binders	A feed-forward neural network with inputs describing structural and structure-based energetic features of 9-aa in peptide sequence and peptide-HLA complex. Structural and energy features are those comprising Talaris 2014 energy function or derived from Table S3 (109)	Structural and energic features: van der Waals interaction, hydrophobic solvation, Coulombic potentials, hydrogen bond energies, side chain rotamer energies, and solvent accessible surface areas (SASA)

In order to predict antigens with high potential for cross-reactivity and off-target toxicity, Jaravine et al. developed Expitope 2.0 that allow analysis of tissue-specific gene expression pattern and prediction of potential side effect in normal tissue, with the ultimate aim of selecting a safer pool of vaccine targets for personalized immunotherapy (89, 111). Zhang et al. applied tetramer-associated T cell receptor sequencing (TetTCR) to resolve up to five cross-reactive peptides per cell and identified patterns associated with TCR cross-reactivity (6). Similarly, Bentzen et al. utilized experimental data and developed an algorithm named Find Individual Motif Occurrence (FIMO) software to create a priority score inferring the likelihood of cross-recognition (5). From each Shannon logo, cross-reactive peptides were predicted from corresponding position-specific scoring matrix (PSSM) using FIMO, and the human proteome was searched for sequences that match each logo.

### Discriminative Features Governing TCR:pMHC Interaction

**Figure 1:**
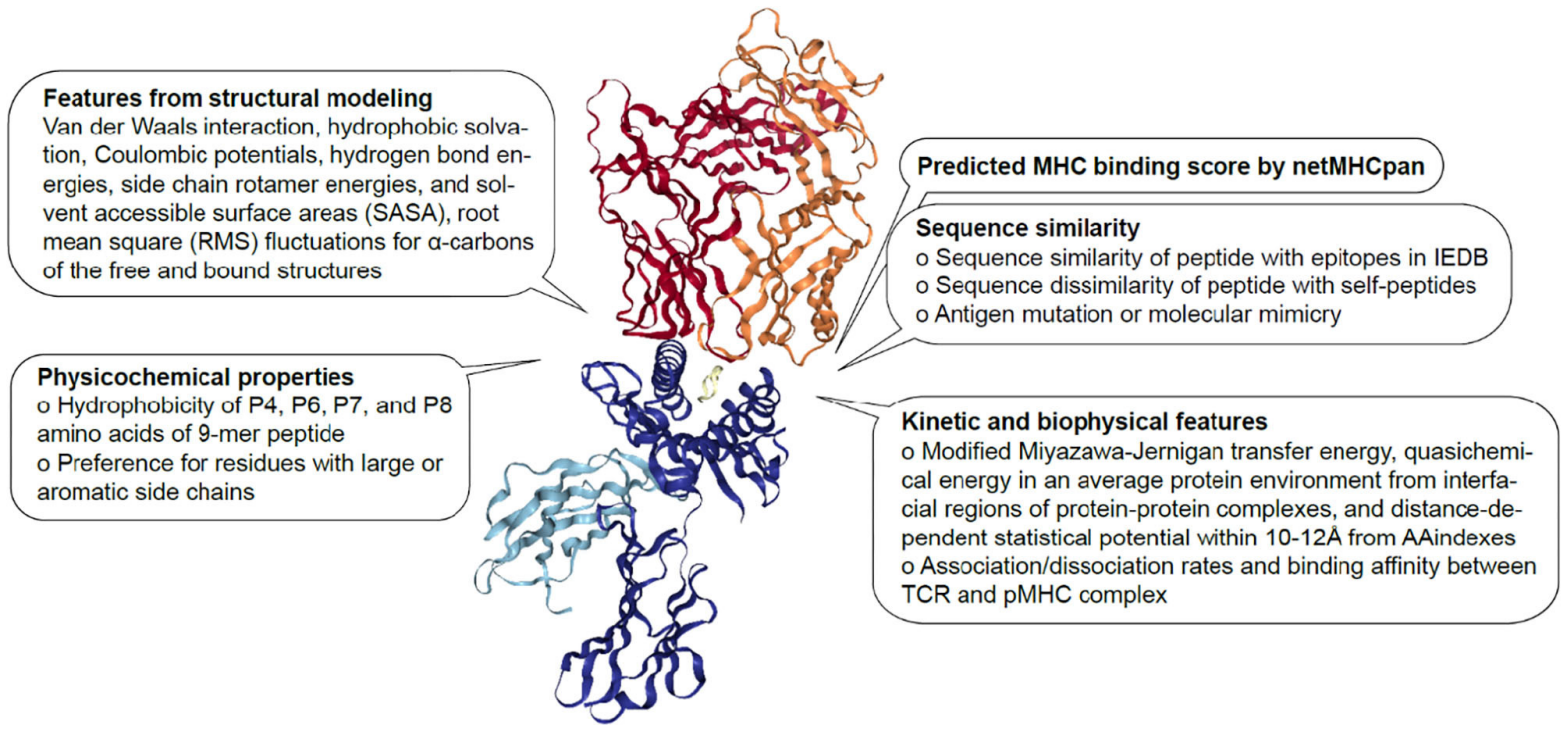
Features associated with TCR:pMHC interaction. Description of sequence-based, structural, kinetic, and biophysical features previously found to be associated with pMHC recognition by TCR The diagram is 1G4 TCR bound to NY-ESO-1/HLA-A*02:01 (PDB 2BNR) where TCRα, TCRβ, MHC, β2-microglobulin and peptide are colored in orange, red, blue, light blue, and yellow, respectively.

Although there have been several attempts to predict immunogenicity, the dual nature of the peptide-specific TCR recognition interface, comprised of both peptide and MHC, makes predicting interaction between TCR and pMHC uniquely challenging. While much of T cell specificity is determined by the promiscuous peptides due to a relatively invariant interaction with MHC molecule (112, 113), it has been demonstrated that TCR:pMHC recognition is influenced by peptide length, physicochemical properties, amino acid sequence especially at central and anchor residues, MHC haplotype and structural landscape (114). Over the years, TCR:pMHC interactions have been extensively studied, thus providing a wealth of data for modeling to be performed from different perspectives (Figure 1). In the following subsections, we will describe a number of discriminative features shown to associate with immunogenicity.

#### Biophysical and Kinetic Features

In addition to the discovery of hotspot residues through TCR sequence alignments (16), biophysical studies revealed that some interactions at the pMHC surface seem more important in triggering a T cell responses (112, 115). This raised a hypothesis that even TCRs sharing a similar TCR footprint may have their unique “interaction profile” (38), and claimed that while conventional hotspots were attributed to amino acid residues, the concept of hotspot should be expanded to account for interaction features, such as hydrogen bonds, van der Waals forces, pockets and coordination of water molecules (115, 116).

A collective effort has identified biological and physical parameters that modulate TCR:pMHC engagement and T cell response [reviewed in (117)], which include binding affinity, association and dissociation rates, half-life of interaction, changes in heat capacity, competition for CD3 molecules and conformational adaptability of TCRs (117–132). Taking these biophysical and kinetic features into account may help to effectively reflect the two-dimensional (2D) and dynamic *in vivo* system whilst modeling TCR:pMHC interactions.

Although we are not currently in the position to perform an *ab initio* prediction based on biophysical and/or structural data, recent studies have started to incorporate physical and chemical properties in developing a generalized TCR scoring function. In a multi-linear regression model, Riley et al. utilized 16 full-atom Rosetta terms (133, 134) and six flexibility terms as predictor variables and experimental binding energies as response variables to estimate the effect of point mutations on thermodynamic stability of the TCR. They identified six significant features, 4 structural terms (van der Waals attractive and repulsive forces, solvation energies and sidechain hydrogen bonding) and 2 flexibility terms (root mean square (RMS) fluctuations for α-carbons of the free and bound structures) contributing to improved performance of the scoring function.

In another application, Haider et al. aimed to engineer an affinity enhanced A6 TCR targeting Tax peptide/HLA-A2 complex (135). They created a set of 219 fitted scoring functions using kinetic and potential energy terms and identified a function (named ZAFFI score) best capable of reproducing affinity changes upon 648 mutations on ovomucoid turkey inhibitor molecule. This work was followed by Pierce et al. identifying an improved scoring function (ZAFFI 1.1) having a higher correlation for a set of DF5 point mutations (136). ZAFFI 1.1 includes six terms: van der Waals attractive and repulsive components, desolvation, intra-residue clash, hydrogen bonding and Coulombic electrostatic force.

In a recent review, Spear et al. have highlighted the significance of considering the previously unappreciated complex relationship between kinetic, cellular and structural patterns that modulate antigen specificity and TCR cross-reactivity in designing TCRs (117). Likewise, such parameters should be taken into account in modeling TCR:pMHC cross-recognition propensities as well as antigen specificity.

#### Features From Structural Modeling

The 3D crystal structures of T cell receptor and their cognate pMHCs have been resolved and deposited in protein database (PDB) (137). The structural T cell receptor database (STCRDab) contains >340 PDB entries and >480 αβTCR structures and facilitates analysis and visualization of TCR structures as well as individual CDR loops (138). This database includes information about MHC type, antigen specificity, Vα-Vβ pairing, orientation, dissociation rate (Kd) and CDR type. Additionally, TCR3d provides information on germline gene usage, antigen binding mode and interface features (139).

Based on the cognate peptide, MHC and TCR structures in the aforementioned database, there have been a number of attempts to accurately predict peptide-MHC conformations, including docking algorithms (140, 141), protein threading (142), all-atom molecular dynamics (MD) simulations (143–145), energy minimization (146) and hybrid of these approaches (147). Likewise, approaches to model pMHC-TCR include MD or Monte Carlo simulations, TCR:pMHC hydrogen bond network analysis (148, 149), binding free energy simulation (150) and CDR loop characterization (130). Both rigid and flexible docking protocols have been proposed to assemble unbound structures (151).

The features retrieved from structural modeling were utilized to predict TCR:pMHC complex formation (151, 152). Mendes et al. applied electrostatic potential and topography data to conduct structure-based prediction among viral epitopes. By using structural features as input for a multivariate statistical model, they showed that use of accessible surface area (ASA, Table 3) adds value to infer immunogenicity and cross-recognition potential. Similarly, Riley et al. showed that hydrophobic SASA and hydrophobic solvation energy values at peptide positions 5, 7, and 8 were in the top 10% of all weights in the neural network for predicting immunogenicity (109).

Recent structural studies have emphasized the importance of structural and physicochemical homology in T cell receptor cross-reactivity (112, 153–160). For example, screening libraries of ligands against 2B4 and 42F3 TCRs revealed that peptides containing sequence motifs at specific positions were found to participate in similar TCR contact networks (112, 153). Collectively, the shared peptide conformation and core residues were shown to limit structural diversity and facilitate cross-recognition.

However, Riley et al. questioned the notion that the pools of ligands for a given TCR is built around core regions of restricted structural and chemical space, and showed that T cell receptors can also cross-react between ligands with little structural or physicochemical commonalities. They demonstrated that the DMF5 TCR can cross-react with divergent antigens by unanticipated rearrangements in peptide and presenting MHC molecules, including binding-induced peptide register shifts. Although dramatic rearrangements did not translate into molecular mimicry, this TCR was capable of cross-reacting with distinct classes of epitopes. Likewise, cross-reactivity has been observed from unrelated pathogens even with a low level of structural homology (31, 33, 68, 69, 161, 162).

These findings suggest that while structural homology may inform cross-recognition potential of peptides having the same structural configuration, current methods are suboptimal in predicting polyspecificity across different classes. Moreover, amino acid mutations at positions distant from direct recognition sites may also have a substantial effect on TCR:pMHC interaction e.g., change in binding parameters and/or structural conformation, and can only be validated by experimentation (163). Altogether these may imply an immense breadth of promiscuity beyond our expectations based on current understanding.

### Elements to Consider in Modeling Immunogenicity or Cross-Reactivity of TCRs

Given the limitations in the current methods to reflect and predict TCR:pMHC recognition, here we describe a few considerations to make in building algorithms to predict immunogenicity or cross-recognition potential.

First, a key challenge in developing machine learning and statistical models to predict immunogenicity is the lack of true negative datasets for TCR-epitope interaction as well as cross-reactivity information. Several groups tackled this limitation by simulating a background or negative data (93, 96, 97, 164). Jurtz et al. approached the problem by creating incorrect combinations of TCRs and peptides i.e., linking TCR sequences with a random peptide different from the cognate target, and produced a balanced set of positive and negative data. Alternatively, Ogishi et al. retrieved the latest set of all characterized peptides and examined coexistence of positive and negative assay results to classify immunogenicity in a population-level (96). Given the limited coverage of cross-reactivity spectrum, a rational simulation would supplement the true negative data for training a classifier.

Additionally, the existing datasets are in a binary format of being immunogenic or non- immunogenic, whereas it is evident that the T cell response is a continuum and comes in different flavors from a mild to a very strong response and varies in functional outcomes such as differential cytokine production. Quantitative T cell response measures associated with each epitope will open a new avenue for rigorous modeling.

Second, current distance measures are mainly context specific and do not capture the true immunogenic capacity of the input peptides. For example, Grouping Lymphocyte Interactions by Paratope Hotspots (GLIPH) and TCRDist that are aimed to detect common antigen specificity groups may not be effective in estimating breadth and/or constituents of the cross-reactome. Cancer specific immunogenic neoantigens that are used for cancer vaccine targets are mainly different from the wild type by only a single point mutation. Engineered affinity-enhanced TCRs have recently been shown to generate unpredicted cross-reactivity even by a single amino acid substitution (64, 165, 166). As such, a naive sequence-based metrics such as Euclidean distance may pose limitation and thus development of a distinct distance metric for evaluating cross-recognition potential may be required.

Third, there is a considerable heterogeneity in the experimental methodologies employed in assessing T cell responses. Although standardizing T cell assays into a single readout is practically difficult, accuracy of predictive algorithms may be enhanced by reflecting the sensitivity and specificity of assays employed for characterizing each epitope.

Fourth, up to date, exhaustive screenings have been performed based on an assumption of invariant MHC interaction. However, previous studies suggested the ability of a TCR to recognize peptides bound by non-canonical HLA molecules (167, 168). In addition to cross-reactivity of virus-specific T cells to HLA-A and -B molecules, van der Zwan et al. reported cross-reactivity of HLA-B^*^08:01-restricted EBV-specific T cell against HLA-C^*^01:02 (169). From a clinical perspective, a severe off-target toxicity was reported by adoptive cell transfer of T cells targeting HLA-A^*^01:01 MAGE-A3 complexes by binding to a Titin-derived peptide displayed on HLA-A^*^01:01 (55). Thus, it may be necessary to screen for peptides bound on non-predicted HLA alleles to project the complete scope of cross-reactivity, and we need to keep in mind that the current sets of data may only reflect the tip of an iceberg.

Lastly, we need to keep in mind that while TCR:pMHC interactions exhibit a remarkable capacity of discrimination, they are often sloppy and cross-reactive. Nevertheless, as exemplified by thymic selection, weaker affinities play an essential role in underpinning the sensitive detection of a wide range of cognate antigens yet keeping it well-balanced from self-reactivity (170, 171). Moreover, low and high-affinity T cells may involve in biological processes differently in regards to e.g., effector and memory differentiation, metabolic reprogramming, and immune response in specific conditions (172, 173). Given the dynamic nature of weak interactions and their potential functional implications, we may need to divert from lessons learned from well-optimized interfaces, such as antigen-antibody binding. We should note that due to challenges involved in measuring low affinity interactions, existing data may be biased in favor of high affinity interactions (174), and may also need to reexamine scoring functions and parameters to reflect dynamic interplay of low and high-affinity T cells for an efficient immune response (175).

## Predicting Common Specificity Group of T Cell Receptors

The amino acid sequence of paired TCR defines its binding specificity. However, we still do not have a full understanding of the mechanisms underpinning the recognition of pMHC complexes by their cognate TCRs. In the last few years, there have been mathematical and computational efforts to find systematic ways to cluster TCRs based on their likely antigen specificity, a phenomenon known as defining common antigen specificity groups.

To identify TCRs specific to a given antigen, one will require to sort and sequence naïve and antigen experienced T cell repertoires. Recent advances in both bulk and single cell sequencing technologies facilitates generation of such datasets in a high throughput manner. A dedicated set of algorithms and software tools will allow computational biologist to further analyze and profile TCR repertoires (176–178). This includes MIXCR and IMGT V-QUEST for assigning raw sequence reads into VJ genes and CDR3 sequences, and VDJtools (179) to compute VJ gene usage statistics as well as repertoire diversity.

Such complementary biological assays and computational platforms enabled robust generation and analysis of millions of TCRs in a single experiment. Importantly, the curated sequences have been deposited in databases such as VDJdb (180) and McPAS-TCR (181). The VDJdb contains >60,000 TCR specificity records associated with their epitope and MHC, and McPAS-TCR holds >5,000 TCRs associated with pathogenic conditions e.g., pathogen infection, cancer and autoimmunity.

**Figure 2:**
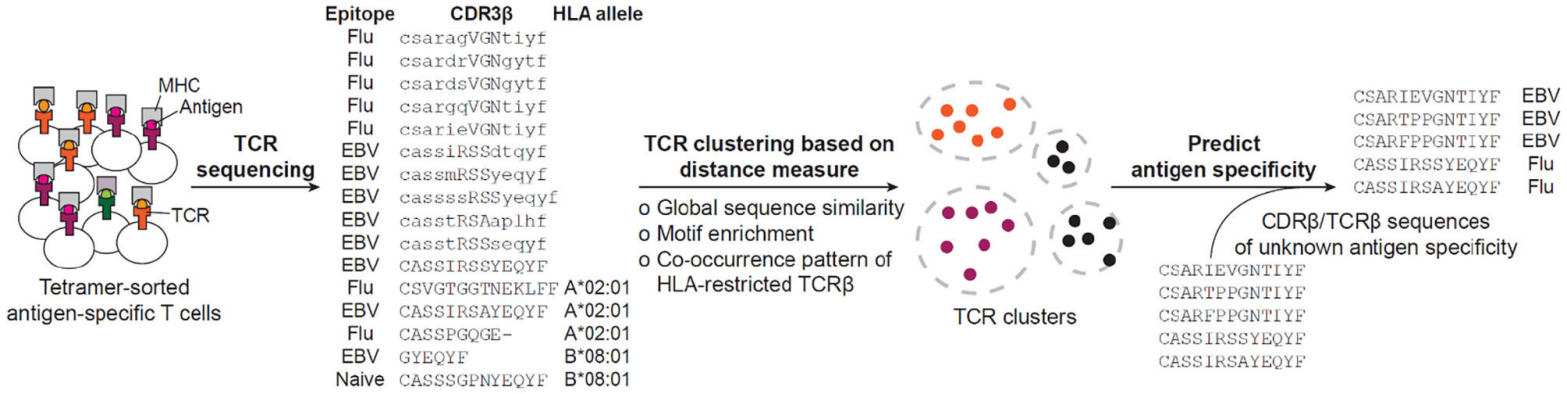
Current workflow for predicting antigen specificity of TCRs. The tetramer-sorted antigen specific CDR3β or TCRβ are clustered by distance measure defined by either global sequence similarity, motif enrichment or sequence co-occurrence pattern. Then, specificity clusters are investigated for their descriptive features, such as enrichment of common V-genes, CDR3 length, clonal expansions, and motif significance, to be considered in making the prediction of antigen specificity. Based on the collection of identified features, previously uncharacterized CDR3βs or TCRβs are predicted for their antigen specificity. The example sequences have been retrieved from (16, 182).

The accumulation of so many antigen-specific TCR sequences, on one hand, urged the development of systematic methods to group TCR sequences according to, for example, their shared antigen specificity, and on the other hand, opened an opportunity to conduct in-depth characterization of antigen-specific TCR repertoires, find shared and conserved features and develop a distance measure that permits clustering and visualization of the TCR space (Figure 2). In the following subsections, we will be looking into a number of such methods.

### Algorithms to Predict Antigen-Specificity of TCRs

The above mentioned rationales have formed the foundation for several recent studies trying to predict specificity groups of TCRs based on their TCR or CDR sequences (15–18, 182–187) (Table 2). By analyzing the collection of TCR sequences, researchers have tried to identify shared features among antigen-specific TCRs and to develop a distance-based classifier capable of assigning previously unobserved TCRs to characterized repertoires. Here are examples of different approaches employed to predict common specificity groups of TCR.

**Table 2:** Algorithms to predict antigen specificity of TCR repertoire.

**References**	**Data**	**Distance measure**	**Clustering algorithm**
Thomas et al. (183)	CDR3 sequences of CD4+ T cell repertoire before and after immunization	Replace each CDR3 by all possible n-mer peptides, then convert each n-mer peptide into numeric Atchley vectors	K-means clustering of Atchley vectors, count number of Atchley vectors assigned to each cluster, and generate into a feature vector. Classify the feature vector using hierarchical clustering (unsupervised) or support vector machine (supervised)
Dash et al. (15)	pMHC-facing loop between CDR2 and CDR3 and trimmed CDR3 sequences from 4,635 paired TCRαβ sequences	Similarity-weighted mismatch distance between the potential pMHC-contacting loops of two TCRs, defined by BLOSUM62 (named TCRdist)	Sampling density nearby each TCR estimated by weighted average distance to the nearest-neighbor receptors in repertoire (a small nearest-neighbor distance, NN-distance). Each TCR repertoire clustered using “greedy” fixed-distance-threshold clustering algorithm. At each step, TCR with the largest number of neighbors within the distance threshold chosen as a cluster center and iterated for all TCRs
Glanville et al. (16)	CDR3 from 5,711 TCRβ sequences	Global similarity by CDR3 hamming distance between two TCRs with same Vβ segment and same-length CDR3. A fold-change enrichment of local convergence motif by observed frequency of the motif over expected frequency in repeat random sampling from naïve distribution	Cluster TCRs sharing either global similarity below Hamming distance threshold (differ <2 amino acids) or share a significant motif (>10-fold enriched and <0.001 probability of occurring than in naïve TCR pool)
Cinelli et al. (184)	CDR3 from CD4+ TCRβ sequences before and after immunization	CDR3β sequences deconstructed into k-mers, then motifs ranked according to one-dimensional Bayesian classifier score comparing their frequency in repertoires of two immunization classes	Top ranking motifs selected and used to create feature vectors to train a support vector machine for classifying into distinct clusters
Priel et al. (185)	~360,000 TCRβ sequences from (188)	Levenshtein distance between TCRβ and cluster representative	UClust algorithm (189). Sort sequences according to their length, then iteratively checks for existing cluster to associate the next sequence whose Levenshtein distance from cluster's representative is smaller than a given threshold to generate “Clone-Attractors” (CAs) network
DeWitt et al. (182)	TCRβ sequences from 666 healthy individuals from (190)	Co-occurrence of global TCRβ (for genetic background) and HLA-restricted TCRβ (for immune history and receptor specificity) by analysis of covariation and hypergeometric distribution to assess significance	DBSCAN algorithm (191) to cluster public TCRβ by occurrence patterns, with (i) predefined similarity/distance threshold and (ii) minimum number of neighbors for a point to be considered as a core
Meysman et al. (186)	Two independent datasets of 412 TCRβ from [(15)] and 2,835 TCRβ sequences	Investigated length-based distance, GapAlign score, profile score, trimer score, dimer score, Lavenshtein distance score, and VJ edit distance	DBSCAN algorithm (191), an unsupervised clustering to group TCRs based on a fixed distance defined in advance
Pogorelyy and Shugay (17)	CDR3 from TCRβ sequences from (190)	Hamming distance, allowing single substitution	TCR similarity networks by Hamming distance and identify enriched TCR network hubs by testing neighborhood size (degree) enrichment against VDJ rearrangement model using ALICE algorithm (192) or against control dataset using TCRnet
Thakkar and Bailey-Kellogg (187)	CDR3 sequences, CDR3α and CDR3β analyzed separately	Local alignment using Smith-Waterman (SW) algorithm with BLOSUM45	Hierarchical agglomerative clustering, with CDRdist (a nearest neighbor classifier to predict label of another CDR based on nearby labeled CDRs) as a comparison function. Clusters defined by CDRdist thresholds
Zhang et al. (18)	82,000 CDR3 sequences from 9,700 tumor RNA-Seq samples from TCGA	Pairwise alignment score with BLOSUM62, normalized by the length of longer CDR3 sequence	From pairwise score matrix, apply a predefined cut-off value (default 3.5) to filter out low scoring comparisons A depth-first search (DFS) on the matrix to identify all connected CDR3 clusters (named iSMART)

**Table 3:** Glossary.

**Term**	**Definition**
Accessible surface area	Also known as solvent-accessible surface area (SASA); the surface area of a biomolecule that is accessible to a solvent. Measurement is usually described in units of square Ångstroms
Adoptive T cell transfer	A type of immunotherapy in which T cells are given to a patient to improve immune functionality to fight diseases
Amino acid index database (AAindex)	A database of amino acid indices and amino acid mutation matrices. An amino acid index is a set of 20 numerical values representing various physicochemical and biochemical properties of amino acids. An amino acid mutation matrix is generally 20 ×20 numerical values representing similarity of amino acids
Clonal expansion	A process in which a small number of precursor cells recognize a specific antigen, proliferate into expanded clones, differentiate and acquire various effector and memory phenotypes
Combinatorial peptide library	A library typically comprised of millions to billions of random peptides covering possible combinations of amino acids in each position
Degeneracy	Ability to recognize diverse ligands
Electrostatic potential	The amount of work needed to move a unit of charge against an electric field
Featured peptide	A peptide with solvent-exposed, prominent side chains or harmonious bulged confirmations and typically correspond to a diverse repertoire of TCRs
Find Individual Motif Occurrence	A motif-based sequence analysis tool that scans a set of sequences for individual matches to each of the motifs provided by the users
Flexible docking	A macromolecular docking where the internal geometry of the interacting partners can be changed when a complex is formed
Heterologous immunity	An immunity that can develop to one pathogen after a host has had exposure to non-identical pathogens
Immunodominant peptide	A peptide having a strong affinity for binding with HLA and for stimulating a T cell response
Kidera factor	A set of orthogonal physicochemical properties that reflect 20 amino acids, which include helix/bend preference, side-chain size, extended structure preference, hydrophobicity, double-bend preference, partial specific volume, flat extended preference, occurrence in alpha region, pK-C and surrounding hydrophobicity
Molecular mimicry	A phenomena that sequence similarities between foreign and self-peptides are sufficient to trigger cross-activation of autoreactive T cells by pathogen-derived peptides
Peptide-MHC display system	A platform with engineered functional peptide-MHC complexes for high-throughput screening of immunogenic peptides against TCRs
Polarization	A process to adopt different functionality in response to the signals from their microenvironment
Positional specific scoring matrix	An amino acid scoring matrix in a 20 ×20 table such that position indexed with amino acids e.g., position (X, Y), gives the score of alignment or substitution of amino acid X with amino acid Y
Private TCR	A TCR unique to an individual
Public TCR	A TCR shared among different individuals
Rigid docking	A computational modeling of the quaternary structure of complexes formed by two or more interacting biological macromolecules, where the relative orientation of interacting partners was allowed to vary but the internal geometry of each of the partners was held fixed
Rosetta terms	A set of 19 terms comprising Rosetta Energy Function 2015 (REF15), a model parametrized from small-molecule and X-ray crystal structure data, used to approximate the energy associated with each biomolecule conformation
Tetramer-associated T cell receptor sequencing	A method to link TCR sequences to their cognate antigens in single cells at high throughput manner. Peptide-TCR binding is determined using a library of DNA-barcoded antigen tetramers
ZAFFI score	Abbreviation for Zlab affinity enhancement; an algorithm to predict the effect of point mutations on binding affinity of TCRs. Training of energy function was performed using a dataset of systematic point mutations at 10 positions on the ovomucoid turkey inhibitor (OMTKY) molecule in four enzyme-inhibitor complexes. The optimal terms and weights for the function was obtained to fit the energies of OMTKY point mutants and tested using point mutations of T cell receptor. The terms and weights making up the score are: van der Waals attractive (0.24), van der Waals repulsive (0.017), Lazaridis-Karplus solvation (0.24), intra-residue clash (0.073) and atomic contact energy (0.32)

#### Co-occurrence Pattern of TCR Sequences

While TCRs are rarely cross-reactive across HLA haplotypes (193), they can be highly promiscuous to different peptides presented on the same HLA (24, 112, 113) and this invariant interaction between TCR:pMHC also confers T cell specificity. Based on this principle, a recent study by DeWitt et al. showed that despite the diversity and complexity of TCR repertoire and pMHC, there exist common patterns across individuals exposed to the same disease. They leveraged this finding to cluster TCRs by their co-occurrence pattern, associated TCR clusters to HLA (i.e., HLA restriction) and predicted antigen specificity of the TCR cluster (182). Using repertoire sequencing data coupled with high-resolution MHC genotyping, they demonstrated striking imprints of common pathogens and clusters of co-occurring TCRs that may represent markers of shared immune exposure.

#### CDR3β Sequence Similarity

As a result of somatic recombination, TCR sequences produce three complementary determinant region (CDR) loops, where CDR1 and CDR2 of α- and β-chains are conventionally believed to govern the interaction with an MHC molecule, and hypervariable CDR3α and CDR3β loops to guide specific engagement of TCRs with MHC-bound cognate peptides (194, 195). A number of studies have observed structural rearrangement of CDR loops during TCR:pMHC interaction. The range of motion is between 0.3 and 11.4Å, where CDR3 loop generally undergoes the largest shifts (196).

Based on the understanding of CDR loops with pMHC interaction, some progress has been made in predicting specificity groups of TCRs based on the similarity of short stretches of TCR amino acid sequences, known as motifs, mainly within CDR3 region (15, 16, 18, 93, 192). Glanville et al. aligned amino acid sequences of all reported TCR:pMHC crystal structures and identified stretches of three to five contiguous amino acids at specific positions in TCRβ CDR3 to be positioned within 5Å of peptide residues. Building upon this finding, they sorted Epstein-Barr virus (EBV), cytomegalovirus (CMV) and influenza-specific T cells, performed single cell sequencing of isolated TCRs or bulk TCRβ sequencing, then again observed similarity in short sequences of CDR3s within hundreds of antigen-specific T cells. The authors proceeded to incorporate these observations into an algorithm for Grouping Lymphocyte Interactions by Paratope Hotspots (GLIPH) that allowed them to cluster TCRs with comparable levels of specificities. Along with GLIPH, several algorithms have recently been proposed such as TCRDist (15) and TCRnet (17), which also relies on CDR3s to cluster TCRs based on the amino acid sequence similarity.

### Improving Accuracy of TCR Specificity Group Prediction

Although current algorithms have been applied in multiple biological contexts such as Alzheimer's disease (197), narcolepsy (198), and PD-1 blockade treatment (199), recent studies reported suboptimality of the algorithms (18) given the limited number of crystal structures concentrated around a few frequently observed viral antigens. Here we present several elements that may facilitate improvement of predictive accuracy.

#### Extending Current Algorithms From CDR3β Amino Acids

A number of recent studies have suggested that integrating information across all six CDRs, instead of considering CDR3α or CDRβ independently, would likely yield a higher performance (15, 16, 182). In particular, Lanzarotti et al. evaluated TCR target prediction models based on incorporation of full TCR paired sequences, 6 CDR loops and/or structural similarity (200). The best performing model was the one incorporating all CDR1, 2, 3 α and β information with greater weight given to CDR3αβ, plus adding structural information (root mean square deviation, RMSD) moderately but consistently improved the performance. Of interest, placing greater weight to CDRβ sequences over CDRα led to decreased predictive power compared to even the flat model. Thus, developing a distance measure that incorporates all CDR1, 2, 3 α and β sequences is likely to demonstrate a higher predictive performance than the current TCR specificity group algorithms.

In addition, translating CDR amino acid sequences into their physicochemical properties and using their inherent properties to cluster TCRs into specificity groups may bring another step forward. Ostmeyer et al. developed a statistical classifier of T cell receptor repertoire that distinguishes tumor tissue from patient-matched healthy tissue of the same organ (201). The classifier was based on physicochemical motifs in CDR3 of TCRβ chains. Here, 4-mer amino acid sequences were represented by their physicochemical properties using Atchley factors—polarity, secondary structure, molecular size/volume, codon diversity and electrostatic charge—and achieved classification accuracy of 93 and 94% for colorectal and breast cancer, respectively.

From previous efforts to reduce dimensionality of a large number of possibly co-linear amino acids properties into small number of orthogonal properties that maintain most of the information contained in the original set, physicochemical properties of amino acids have been characterized and summarized into e.g., 10 Kidera factors (202) and 5 Atchley factors (203). Analyzing occurrence of “physicochemical motifs” in TCRs along with structural features e.g., RMSD will bring one step closer to accurately identifying TCR specificity groups.

#### Application of Single Cell Technologies for Paired TCRαβ Profiling

While bulk TCR sequencing revolutionized characterization of TCR repertoire in different pathological settings e.g., tumor immunology and autoimmunity (204), β chain analysis has always been the main target due to its higher combinatorial potential and its ability to represent as “unique label” for a T cell after allelic exclusion. However, many studies highlighted the pairing of α and β chain to reflect biological function of a T cell *in vivo* (205, 206) and that even α chain alone can accurately differentiate T cell subsets by its function and phenotype (207, 208). Izraelson et al. narrowed TCR complexity by fixing TCRβ background and thus allowing TCR diversity and antigen specificity to be determined by TCRα chain alone. Then, using a similarity measure, R metric describing the correlation of overlapping clonotype frequencies, could “digitally” differentiate their TCRα repertoire from spleen, thymus and lymph nodes into functional T cell subsets of T_reg_, T_eff_, and naïve CD4 T cells. This illustrates that while TCRβ may operate as “unique label” of a T cell, TCRα may as well encode essential information about its phenotype, function and specificity.

Recent advancement in single cell approaches opened the door for elucidating how particular α-β pairing contributes to antigen specificity. In particular, several groups have started to implement single cell platforms for simultaneous identification of TCRαβ sequence and antigen specificity in a high-throughput manner across multiple pMHCs (5, 6, 209). For example, Bentzen et al. applied a large library of >100 DNA barcode-labeled MHC multimers to stain antigen-specific T cells, isolate T cells bound by MHC multimers using flow cytometry followed by a droplet-based single cell sequencing to capture αβTCR transcripts and the MHC-associated DNA barcodes in parallel.

The potential benefits of identifying TCR αβ pairs coupled to antigen specificities include but are not limited to: (i) identifying unique CDR3 α/β signatures dictating epitope recognition for possible applications across the field of adaptive immunity e.g., efficient design of TCRs for vaccine development or targeted immunotherapy (210), (ii) portraying T cell ancestry in response to pathogen exposure, (iii) investigating which functional T cell subsets have undergone clonal expansion in response to different antigens, (iv) examining distinct phenotypic and functional properties of T cells responded to different antigens, and (v) identifying TCRαβ heterodimers losing functional integrity *in vitro*, which will be useful for therapeutic applications (211).

Importantly, the large number of paired TCRαβs coupled to antigen specificity can be fed into computational models improving accuracy of prediction. The exhaustive list of recognition patterns combined with increasing structural information about TCR:pMHC interaction will assist prediction of specific TCR:pMHC interaction based on TCR sequence (10). Of interest, with increasing reports focusing on TCR repertoire of antigen-specific populations, the latest studies have started to compare predictive performance on different datasets. Thakkar et al. have analyzed repertoires from a twin pair study (212), antigen-specific data from GLIPH and TCRDist studies (15, 16) and pathology-associated data from McPAS-TCR to evaluate the trade-off between sensitivity and specificity of predictive algorithms in different pathology, antigen, MHC restriction settings (187). As discussed by Thakkar et al., while datasets were analyzed individually in the study, integrating multiple datasets and large number of paired TCRαβs should provide insights into common modalities of recognition and broader functional associations across antigens from different pathologies.

## The Interface Between Common Specificity Group and Cross-Reactivity of TCR to Model the Landscape of Interaction Propensities

Despite interest in mapping the TCR:pMHC interactions, a combinatorial approach exploring the mutation space of TCRs against corresponding peptide cross-reactome has not been exhaustively performed. Thus, it would be exceptionally challenging to account for the whole range of available TCRs and surveilling pMHCs.

**Figure 3:**
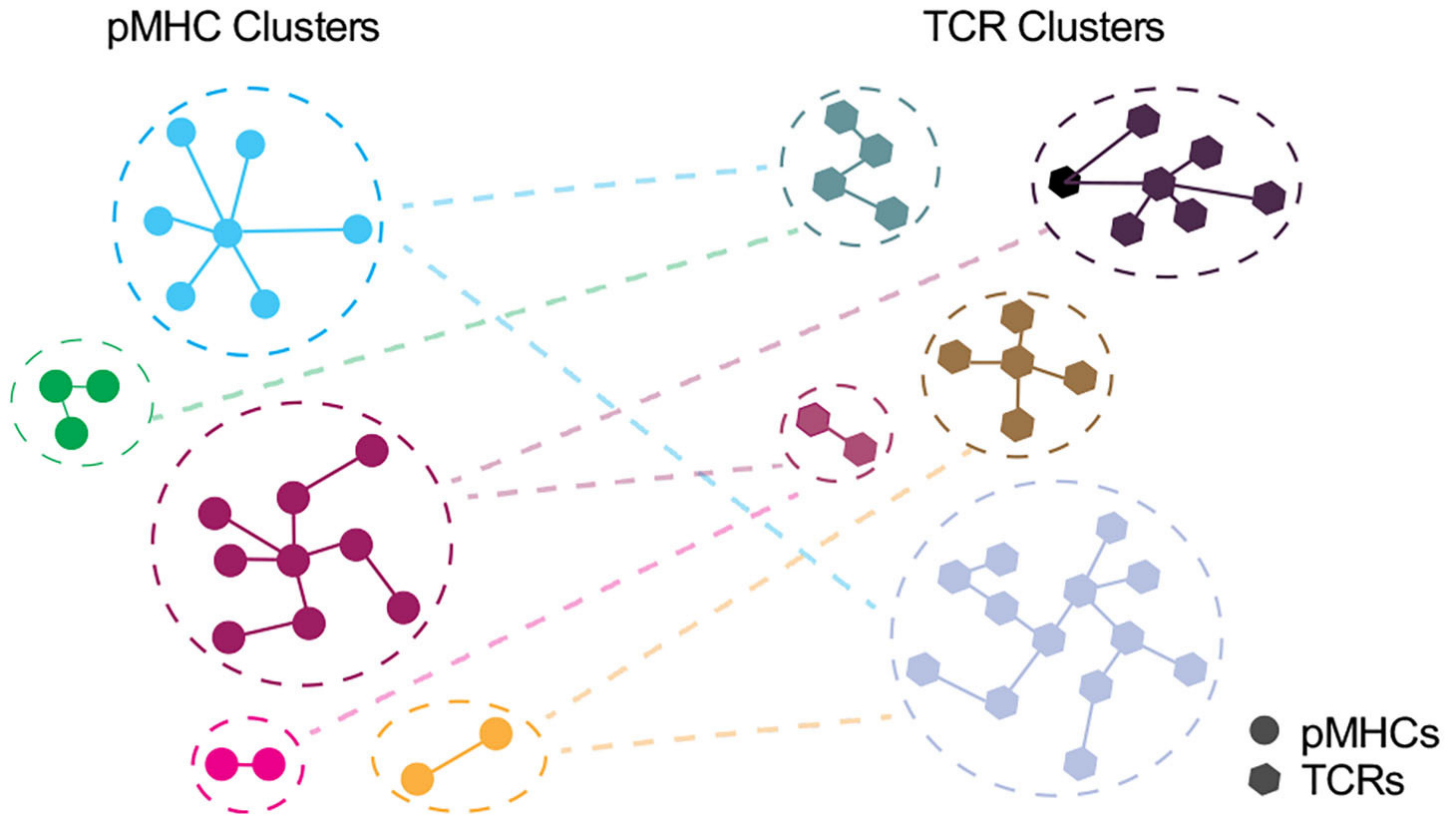
Interplay between unique clusters of pMHCs and TCRs. In an ideal world with an accurate distance measure, pMHCs in the same cluster should share the common specificity toward TCRs and vice versa. Each node denotes pMHC (circle) or TCR (polygon) entities and edge denote the distance with the closest pMHC or TCR, respectively.

Depicting the cross-recognition of TCRs and pMHCs in >10 (6) space, modeling TCR:pMHC landscape should be taken as a cross-talk between unique and representative clusters of pMHCs and TCRs rather than individual entities (Figure 3). However, as discussed by Bradley and Thomas we currently do not know whether TCRs closely related by antigen specificity algorithms e.g., GLIPH or TCRDist are expected to have similar cross-reactome (213). An intuitive answer would be that TCRs with a shorter distance, especially those within the same cluster, will have a greater overlap of cross-reactive peptides. However, as elucidated by structural studies, sequence similarity cannot adequately represent cross-recognition potential as TCRs may have multiple configurations for different classes of epitopes (67).

Therefore, modeling this dynamic interplay may require the development of an accurate distance measure to group TCRs informative of their antigen specificity and/or cross-reactivity. This will require assessment of all identified features, such as paired TCRαβ sequences, n-mer motifs, physicochemical properties as well as structural, physical and kinetic parameters, to derive a minimum set of features with maximum association to immunogenicity. These features will become a toolkit for developing TCR and pMHC distance measures to discriminate >10^6^ TCRs and >10^18^ peptides into designated clusters. Following the classification of clusters, the relationship between TCR and pMHC clusters can further be explored—it may segregate into a linear function or may yield an indistinct pattern where even the repertoires for closely related epitopes have divergent landscape with a very limited overlap.

## Conclusion and Outlook

Here we discuss two fundamental principles of TCR:pMHC interaction, antigen specificity and TCR cross-reactivity. Modeling the underlying principles by cellular, kinetic, and structural features will deepen our understanding on the organizational principle of TCR repertoires.

Recent technological advancements have opened doors for screening antigen-specific TCRs and cross-reactive peptides in a high-throughput manner. In particular, MHC multimer screening in combination with multimodal single cell technologies increased the breadth of T cell analysis by allowing integration of antigen specificity with immune repertoire, transcriptomic and proteomic profiling (7, 19, 20). Further developments in biological systems will provide larger training sets for the *in silico* analyses. These analyses simultaneously give information on: (i) paired TCR sequences, (ii) pMHC specificities across a large epitope library, and (iii) transcriptomics and proteomics measurements of single T cells profiled in parallel. The multi-omics integration will enable in-depth analysis of phenotypic and functional states of each T cell and correlate with their TCR sequences and pMHC interaction.

The present algorithms have not distinguished TCR repertoires by their functional subsets, such as CD4^+^ and CD8^+^ T cells with pro-inflammatory or regulatory functions, largely due to lack of sufficient annotations. Given that each subsets have distinct dynamics according to pathogenic conditions, e.g., viral infection, cancer or autoimmunity, utilization of subset-specific TCR repertoire may further improve predictability of epitope immunogenicity (96). In this regard, the recent efforts to integrate TCR sequencing with transcriptomic and proteomic profiling in a single cell level will enrich present-day datasets (19, 20).

Along with an increasing wealth of experimental and sequencing data, there have been advancements in *in silico* approaches to analyze, model and predict TCR:pMHC interaction. Further efforts will provide insights into specific TCR recognition and organizational principles of the repertoire and support a wide range of applications from discovering potential drivers of allergy, autoimmunity and tolerance (160, 214–216) to identifying cancer neoantigens and developing personalized vaccines (217–219).

For instance, recent studies have focused on a rational computer-aided approach to TCR engineering as a more predictable and safer approach to TCR design (135, 136, 220–222). They used a fine manipulation of structural topography of TCR:pMHC interaction and specific kinetic parameters to better control the potential for cross-reactivity (223). Another study exploited structure-guided computational design of DMF5 TCR by using both “positive design” to enhance peptide-centric binding and “negative design” to weaken interaction with the MHC (136). While the positive design alone introduced new cross-reactivities thus weakened T cell potency, a combination of both positive and negative design maintained the recognition potential whilst cross-reactivity toward other MART-1 homologs was reduced and cross-reactivity against more divergent class of epitopes was eliminated.

Ultimately, building a complete map portraying the TCR:pMHC interface will provide opportunities to describe the response to dynamic interactions in the immune system. The examples include: (i) dynamic changes of antigen-specific TCR repertoire after adoptive transfer (224), (ii) mechanism of molecular mimicry and preferential directionality in antigen specificity, and (iii) influence of private repertoire and immunological history on antigen specificity. Finally, an extensive understanding of dynamic T cell response will allow development of personalized treatments by taking into account the individual's endogenous ability to target a given disease-specific antigen as well as the personal risk of autoimmunity.

## Author Contributions

HK conceived and designed the study. CL conducted literature review. HK and CL wrote the manuscript with contributions from GN, MS, GO, and AS. HK and AS supervised the project. All authors contributed to the interpretation of the observations.

## Conflict of Interest

GO has served on advisory boards or holds consultancies or equity with Eli Lilly, Novartis, Janssen, Sanofi, Orbit Discovery and UCB Pharma, and has undertaken clinical trials with Atopix, Regeneron/Sanofi, Roche. The remaining authors declare that the research was conducted in the absence of any commercial or financial relationships that could be construed as a potential conflict of interest.
